# Oral health status of the disabled compared with that of the non-disabled in Korea: A propensity score matching analysis

**DOI:** 10.1371/journal.pone.0208246

**Published:** 2019-01-14

**Authors:** Jae-Young Lee, Kyung-Cheol Lim, So-Yun Kim, Hye-Ran Paik, Young-Jae Kim, Bo-Hyoung Jin

**Affiliations:** 1 Department of Preventive and Social Dentistry, School of Dentistry, Seoul National University, Seoul, Republic of Korea; 2 Dental Research Institute, Seoul National University, Seoul, Republic of Korea; 3 Department of Consumer Science, College of Human Ecology, Seoul National University, Seoul, Republic of Korea; 4 Department of Pediatric Dentistry, School of Dentistry, Seoul National University, Seoul, Republic of Korea; IRCCS E. Medea, ITALY

## Abstract

**Background:**

There are many types of disabilities, and each type has a variety related to socioeconomic factors. Such factors affect to many health problems of the disabled. However, surveys of the oral health status of the disabled in Korea are rare.

**Objective:**

The purpose of this study was to estimate oral health disparity through comparing oral health status of the disabled to the non-disabled, adjusted for the net effect of the disability on oral health status.

**Methods:**

A cross-sectional study was conducted among the disabled in urban and suburban areas in Korea from June to September 2016. People with physical, mental, and multiple disabilities took part in this survey. The clinical examinations were carried out by trained dentists. Statistical analysis was performed to quantify the association between oral health and socioeconomic status after restricting the analysis using a propensity score matching method.

**Results:**

The disabled had more DMFT, DT, and MT, fewer FT, and fewer teeth than the non-disabled based on entire groups (P<0.01). No difference in the ratio of periodontitis was observed. The subjects with mental disabilities (MD) scored 3.09 (95% CI, 1.07–8.97), and those with multiple disabilities scored 4.37 (95% CI, 1.16–16.37) for edentulous status. The MD had an odds ratio of 1.34 (95% CI, 1.03–1.74), and those with multiple disabilities had an odds ratio of 1.75 (95% CI, 1.11–2.76) for the DMFT index.

**Conclusions:**

These results represent poor oral health status of the disabled compared to the non-disabled. Consequentially, we can verify that not only the existence of disability but also the type of disability has a decisive effect on oral health condition. This comparison is necessary to widen our approach to evaluate the actual status condition of the disabled.

## Introduction

The World Health Organization (WHO) has defined disability as complex, dynamic, multidimensional, and contested. Over recent decades, the disabled people’s movement—together with numerous researchers from the social and health sciences—has identified the role of social and physical barriers in disability [1, 2]. Approximately 15% of the world’s population lives with some form of disability, and there is an annual increment of approximately 5% in the registration of disabled people in Korea [3].

There are many types of disabilities, and each type has different behavioral characteristics and social factors. Due to such behavioral and social factors, disabled people have many medical problems. Notably, multiple factors, including disability type and institutionalization, can affect oral health conditions and disease prevalence [4].

In the past, the oral health status survey of non-disabled people was conducted by the Korean Ministry of Health and Welfare. However, this was assessed the national oral health status through the targeted convenient sampling methods, so it was hard to investigate the cause of oral health breakdown and the necessity for oral health care to grasp the actual state of the residents’ oral health. Therefore, the primary data for oral health policies for the disabled are still lacked in Korea.

The U.S. Surgeon General’s report “Oral Health in America” noted that people living below the poverty level and those with mental or physical disabilities have poorer oral health conditions than does the general population [5]. Many other studies have shown that the disabled also have poor oral health. The oral health of many disabled is more miserable and more limited for the accessibility of to the dental care. Oral disease is a major health problem for adults with disabilities. Poor oral health status has an adverse impact on infection, nutrition, digestion, chewing, appearance, and speech [6]. Socioeconomic disorders affect more people with disabilities than non-disabled people, which makes it hard for them to manage their lives properly. Moreover, because there has been no actual study on these differences in socioeconomic status, appropriate institutional management has been insufficient. Therefore, this research was conducted to obtain primary oral health status data of people with disability, adjusted for the effect of disability on oral health status and using the propensity score matching method.

## Materials and methods

### Study design

A cross-sectional study was conducted with the disabled in urban and suburban areas in Korea from June to September 2016. Seoul is representative uraban in Korea with estimated total the disabled population of 391,753 (4.00%) and Chungju is suburban area in Korea with estimated total the disabled population of 12,961 (6.17%).

The standard of disability categorization was derived by Korean law for people with disability. In Korea, the classification standard of disability complies medical classification based on International Classification of Disease, 10^th^ version (ICD-10). And the disability was hierarchically classified 3 kinds (physical, mental, multiple disability), 4 middle scales (external physical, internal physical, developmental, mental disorder) and 15 subscales. In one of disability kinds, Physical disability include 12 subscales;the physically challenged, brain lesions, visual or hearing impairment, speech impediment, kidney disorder, cardiac lesion, respiratory disorder, hepatopathy, facial disorder, intestinal or urinary fistula, and epilepsy. And mental disability include 3 subscales;intellectual disability, autistic disorder, mental disorder. Multiple disability defined to having 2 complex subscales at least. People with physical, mental, and multiple disabilities took part in this cross-sectional trial survey getting approval by institutional review board at Seoul National University School of Dentistry (IRB No. S-D20160014). The participant or a guardian completed the questionnaire (personal data, oral habits, and other factors, etc.). To analyze comparisons between disabled and non-disabled people, we used data from the Korean National Health and Nutrition Examination Survey (KNHANES) from the years 2007 to 2014.

### Populations

The eligibility matched criteria between oral health survey of the disabled and the Korean National Health and Nutrition Examination Survey were based on similar socioeconomic status by age, gender, education status, and region. From June to September 2016, total number of oral health survey participants with disabilities was 1,729. Excluding missing values, data from 986 participants in this survey and 2,955 non-disabled individuals from the Korea National Health and Nutrition Examination Survey (KNHANES) (N = 10,619) were used in propensity score matching (1:3). The majority were male (73.8%) and mean age at baseline was 44.6 yrs (20 to 89 yrs) in the disabled group and the majority were male (70.9%) and mean age at baseline was 45.7 yrs (20 to 88 yrs) in the non-disabled group. It is possible to access KNHANES raw data from website (https://knhanes.cdc.go.kr/knhanes/main.do) after request authority to acquisition.

### Data collection instruments

Data from the participants was collected through clinical examination guided by well-trained dentist and dental hygienist. The Korean National Health and Nutrition Examination Survey (KNHANES) provides questionnaires to collect data of socio-demographic variables include: gender, age, residence, location and educational background.

### Clinical examinations

The examination was carried out using a portable dental unit equipped with a dental light, mouth mirrors, and periodontal probes. For the assessment of oral health status, all teeth were examined by 4 trained dentists using the World Health Organizationdental caries examination criteria [7] and The decayed, missing, filled teeth (DMFT) is representative variable for assessing dental caries prevalence. DMFT is simply counted the number of decayed, missing (due to caries only) and restored teeth (due to caries only). The Community Periodontal index of treatment needs (CPITN) protocol was used for diagnositic criteria of periodontitis. And periodontitis was defined as community periodontal index 3 and 4;indication probing depth excluded value for inaccessible state. After calibration training of dentists for securing valid data, measured interpersonal mean of kappa index was 0.98.

Periodontal status was recorded at six sites around each tooth (mesiobuccal, mesiolingual, distobuccal, distolingual, and mesial), except for third molars. A CPITN score of 3 and 4 at any site meant that the participant was considered to have periodontitis.

### Statistical analysis

We used the propensity score matching method, defining as the conditional probability of being treated after controlling for covariates, can be used to balance the covariates in the two groups and therefore reduce this bias. As a result, the ultimate goal was to grasp the actual effects of the disability on oral health.

Baseline characteristics were compared between people with a disability and non-disabled people using the standardized difference. A propensity score analysis was performed to account for selection bias in those who did not have a disability, as a bivariate comparison between the disabled and the non-disabled groups showed significant differences across the covariates listed above. In the first step, we performed 1:3 matching to match those who had a disability with those who did not. In propensity score matching, we checked the balance of covariates using the relative multivariate imbalance L1 test [8]. It can be used to balance the covariates in the two groups, and therefore reduce this bias.

As a check of appropriate matching, the disabled were divided by type of disability—physical, mental, or multiple (two or more complex disabilities)—based on regulations for handicapped welfare in Korea. Each group’s characteristics were evaluated for differences between the disabled and the non-disabled using a one-way ANOVA test. Separate odds ratios (ORs) and 95% confidence intervals (CIs) were computed to quantify the association between the disabled and the non-disabled. To quantify the association with oral health outcomes and socioeconomic status, the analysis was restricted by propensity score matching using the R software (R: a language and environment for statistical computing, version 3.2.5; R Foundation for statistical computing, Vienna, Austria) and SPSS (version 24.0; SPSS Inc., Chicago, IL, USA).

## Results

**Fig 1:**
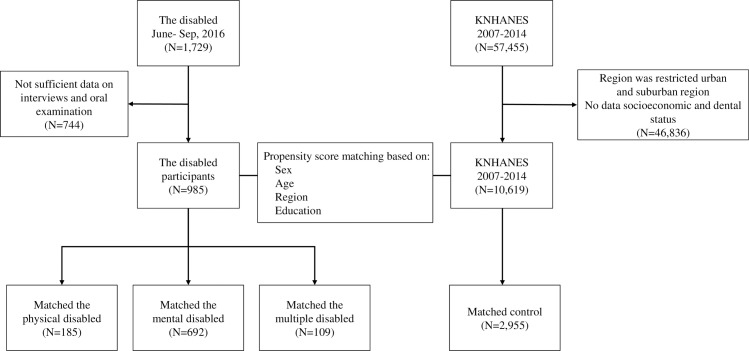
Participant selection diagram. Propensity score matching was applied for each cohort using the covariates gender, age, region, and education. “Multiply disabled” means people with two or more complex disabilities. *KNHANES, the Korean National Health and Nutrition Examination Survey data.

The cohort derivation is summarized in Fig 1; we identified 10,619 non-disabled people for comparison with the disabled (N = 986). Several differences were observed in both groups before the propensity score matching (Table 1). The propensity matching process yielded groups that were well matched based on the characteristics of the verification study (Table 1). No significant differences were observed.

**Table 1:** Propensity score matching was applied for each cohort using the covariates gender, age, region, and education.

Variable	Original cohort			Propensity score matched cohort		
	Disabled, n (%)	Non-disabled, n (%)	Standardized difference	Disabled, n (%)	Non-disabled, n (%)	Standardized difference
N	986	10,619		985	2,955	
Age, yrs, (SD)	39.1 (17.4)	39.5 (22.1)	-0.024	39.1 (17.4)	37.8 (19.7)	0.085
Gender						
Male	697 (70.8)	4,719 (44.4)	-0.578	697 (70.8)	2091 (70.8)	0.000
Female	288 (29.2)	5,900 (55.6)		288 (29.2)	864 (29.2)	
Region						
Urban	771 (78.3)	9,118 (85.9)	0.184	771 (78.3)	2,313 (78.3)	0.000
Suburban	214 (21.7)	1,501 (14.1)		214 (21.7)	642 (21.7)	
Education						
≤Elementary school	312 (31.7)	3,776 (35.6)	-0.117	312 (31.7)	936 (31.7)	-0.007
Middle school	132 (13.4)	1,196 (11.3)		132 (13.4)	396 (13.4)	
High school	452 (45.9)	2,822 (26.6)		453 (45.9)	1,336 (45.2)	
≥College	89 (9.0)	2,825 (26.6)		89 (9.0)	287 (9.7)	

In this propensity score model, we were able to match 986 of the 1,729 in the disabled group to similar controls among the 10,619 non-disabled people in KNHANES (N = 2,955).

After propensity score matching, all covariates were balanced, with the greatest standardized difference being 8.5% and a multivariate imbalance score of 0.214.

### Comparison of oral health status between the disabled and the non-disabled

The disabled had more DMFT, decayed teeth, missing teeth and lower filled teeth than did the non-disabled based on entire groups (P<0.01). Additionally, the disabled in the whole disabled group had fewer teeth than did people without disabilities (P<0.01). The ratio of edentulous patients was higher in the disabled group than in the non-disabled group (P<0.01). However, there was no difference in the ratio of periodontitis patients between the disabled group and the non-disabled group.

**Fig 2:**
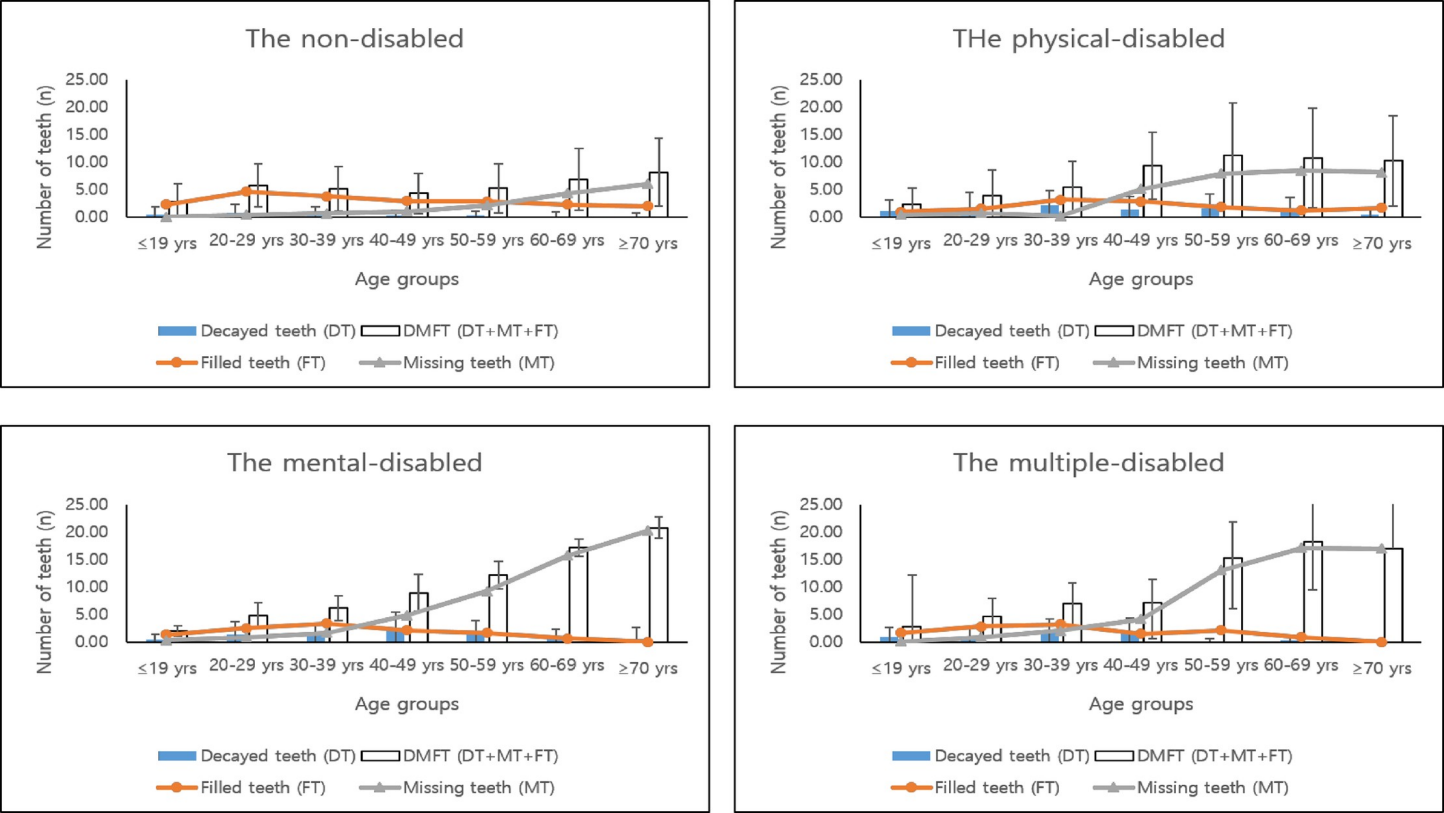
Decayed teeth, missing teeth, filled teeth, and DMFT variation tendency as age increases by disability classification. “Multiply disabled” means people with two or more complex disabilities. (A) Mean number of decayed, missing, filled teeth in permanent teeth of the non-disabled by age. (B) Mean number of decayed, missing, filled teeth in permanent teeth of the physically disabled by age. (C) Mean number of decayed, missing, filled teeth in permanent teeth of the mentally disabled by age. (D) Mean number of decayed, missing, filled teeth in permanent teeth of the multiply disabled by age.

The tendency of DMFT with age increased for both groups. However, non-disabled people had more filled teeth than did the disabled. As shown in Table 2 and Fig 2, we found that the crossing point between missing teeth and filled teeth for the disabled (between the thirties and the forties) was earlier than for the non-disabled (50–59 yrs). Above this age, the slope of missing teeth increased, and the graph for the disabled was steeper than for the non-disabled.

**Table 2:** Oral health status based on present disability.

Variable	Physically disabled	Mentally disabled	Multiply disabled	Non-disabled	p-value
N	185	692	109	2,958	
DT (SD)	1.29 (2.34) *	1.24 (2.48)*	1.03 (1.91) *	0.45 (1.15)	0.000
FT (SD)	1.66 (2.74) *	1.99 (3.10)*	2.07 (3.11) *	3.00 (3.44)	0.000
MT (SD)	4.31 (7.61) *	5.04 (7.91)*	5.06 (8.47) *	1.45 (3.36)	0.000
DMFT (SD)	7.26 (7.62) *	8.26 (7.90)*	8.17 (7.83) *	4.90 (4.49)	0.000
Present number of teeth (SD)	23.18 (7.52) *	23.11 (7.61) *	23.17 (8.09)	24.94 (5.64)	0.000
Edentate status^†^ (n(%))					
Non-edentate	179 (96.8)	664 (96.1)	103 (94.5)	2,926 (99.0)	0.000
Edentate	6 (3.2)	27 (3.9)	6 (5.5)	29 (1.0)	
Periodontal status^‡^ (n(%))					
Healthy	111 (75.0)	438 (73.5)	71 (76.3)	2,055 (74.2)	0.935
Periodontitis	37 (25.0)	158 (26.5)	22 (23.7)	715 (25.8)	

*Multiple comparison testing (Dunnett’s T3) with the non-disabled.

^†^Edentate status excluded value for inaccessible state (age, missing): mentally disabled, n = 1; non-disabled, n = 4.

^‡^Periodontitis was defined as community periodontal index 3–4 excluded value for inaccessible state (age, missing): physically disabled, n = 37; mentally disabled, n = 96; multiply disabled, n = 16; non-disabled, n = 188.

DT, decayed teeth; FT, filled teeth; MT, missing teeth; DMFT: DT+MT+FT; Edentate, having no teeth. “Multiply disabled” means people with two or more complex disabilities.

### Multivariate association among the disabled and established risk factors for dental caries and edentulous status

In a logistic regression model, after adjusting for socioeconomic status, the subjects with physical disabilities had an odds ratio of 1.47 (95% CI, 0.44–4.93; P = 0.54), those with mental disabilities had an odds ratio of 3.09 (95% CI, 1.07–8.97; P = 0.38), and those with multiple disabilities had an odds ratio of 4.37 (95% CI, 1.16–16.37; P = 0.29) for completely edentulous status.

When the 75th percentile was used as the threshold for dichotomization of the outcome variables [9], the physically disabled had an odds ratio of 1.19 (95% CI, 0.81–1.74; P = 0.38), the mentally disabled had an odds ratio of 1.34 (95% CI, 1.03–1.74; P = 0.03), and the multiply disabled had an odds ratio of 1.75 (95% CI, 1.11–2.76; P = 0.02).

## Discussion

In the present study, the types of the disability were shown to be particularly influential factors of oral health, even after propensity score matching and competing for risk adjustment. Mainly, it was confirmed that the prevalence of edentulous loss of teeth and the incidence of dental caries were higher in persons with a disability than in the non-disabled. Finally, we also confirmed that the tooth loss caused by dental caries increased steeply as age increased after the twenties, in contrast to tooth loss in non-disabled people. Some limitations of this study included the sampling process. It is impossible to reflect the oral health condition of all people with disabilities in Korea because we aimed to design and advocate for an oral health promotion program using convenience sampling based on the community level. Therefore, more samples for stratification by type of disability will be needed for a more precise analysis. Types of disability are subdivided into 15 categories by the regulations for handicapped welfare in Korea. Additionally, each type of disability has different behavioral characteristics. Thus, our results may not reflect the extent to which individual comparisons among specific categories may be made. Despite this limitation, this analytic model can be used to compare any type of disability or disease with controls.

The primary findings of the present study are that individuals with disabilities have worse dental caries problems than the non-disabled, regardless of the type of disability (Table 2). In a previous national population survey, the rates of dental caries among the disabled population were found to be higher than in the general population for all age groups studied in the other countries [10, 11]. This result was consistent with findings in India and Italy, Ethiopia [12–14]. Biruktawit [14] reported that people with a mental disorder had demonstrated periodontal pockets and higher DMFT score. Huang reported that people with disability have significantly more missing teeth and fewer restored teeth compared with people with mild disabilities [15]. These indicated that people with disabilities were less likely to acquire appropriate dental treatment needs. Explanations for the poor oral health status of the disabled generate many controversies. Additionally, differences in results are due in part to the fact that the methodologies used have not been the same in many studies, and the level of public welfare for the disabled differs among countries [16]. Notwithstanding, there are some facilities for people with disabilities in Korea. The advantages of policies and medical services for an individual with a disability tend to focus on school age group, and there are also not many systematic oral health promotion programs for people with disabilities.

After people with disabilities leave an institute and become adults, missing teeth from dental caries increase at a rapid rate after the twenties (as seen in Fig 2). However, the problem also occurs in the presence of management, the most commonly cited issue being that the treatment needs of people with disabilities were severely underestimated by both the caregiver and the dentist. The degree of advanced pathology found in the population would suggest that pain suffered was also underestimated because patients with special needs for dental care may be limited by the ability of their caregivers to evaluate their oral condition [17]. This situation shows that when compared with the non-disabled, the disabled display the reversal phenomenon between the filled teeth and the missing teeth earlier, and once they are older than age 30, the disabled show a sharp increase in the number of missing teeth and have a lower number of filled teeth than do the non-disabled (Fig 2). Also, this finding indicates that many people with disabilities do not receive proper dental care once they are adults, which worsens their oral health status. This research also showed that with high-risk dental caries status, the disabled had 1.34 times (mentally disabled) and 1.75 times (multiply disabled) the odds of having DMFT compared to the non-disabled (Table 3).

**Table 3:** Logistic regression analysis for association between disability and dental caries.

	DMFT		DMFT		OR (95% CI)	
Independent variable	≤50^th^ percentile	≥50^th^ percentile	≤75^th^ percentile	≥75^th^ percentile	≥50^th^ percentile	≥75^th^ percentile
Type of disability						
Non-disabled	1342 (45.4)	1613 (54.6)	2191 (74.1)	764 (25.9)		
Physically disabled	74 (40.0)	111 (60.0)	115 (62.2)	70 (37.8)	0.99 (0.69–1.42)	1.19 (0.81–1.74)
Mentally disabled	231 (33.4)	460 (66.6)	400 (57.9)	291 (42.1)	1.13 (0.88–1.44)	1.34 (1.03–1.74)
Multiply disabled	35 (32.1)	74 (67.9)	60 (55.0)	49 (45.0)	1.44 (0.91–2.29)	1.75 (1.11–2.76)
Gender						
Male	1,281 (45.9)	1,507 (54.1)	2,015 (72.3)	773 (27.7)		
Female	401 (34.8)	751 (65.2)	751 (65.2)	401 (34.8)	1.92 (1.64–2.25)	2.02 (1.71–2.39)
Education						
≤Elementary school	646 (51.8)	602 (48.2)	889 (71.2)	359 (28.8)		
Middle school	240 (45.5)	288 (54.5)	397 (75.2)	131 (24.8)	1.37 (1.09–1.71)	0.94 (0.72–1.21)
High school	680 (38.0)	1,108 (62.0)	1,245 (69.6)	543 (30.4)	1.48 (1.22–1.79)	1.07 (0.86–1.32)
≥College	116 (30.9)	260 (69.1)	235 (62.5)	141 (37.5)	1.83 (1.38–2.42)	1.25 (0.94–1.67)
Health security system						
National health insurance*	1,471 (45.1)	1,788 (54.9)	2,404 (73.8)	855 (26.2)		
Medical aid type 1	175 (29.5)	418 (70.5)	295 (49.7)	298 (50.3)	1.35 (1.00–1.82)	1.65 (1.22–2.22)
Medical aid type 2	36 (40.9)	52 (59.1)	67 (76.1)	21 (23.9)	1.14 (0.71–1.83)	0.78 (0.61–0.89)
Region						
Urban	1,264 (41.0)	1,820 (59.0)	2,124 (68.9)	960 (31.1)		
Suburban	418 (48.8)	438 (51.2)	642 (75.0)	214 (25.0)	0.71 (0.60–0.84)	0.74 (0.61–0.89)

Additional independent variables included in this model were age (7 categories) and income (5 categories), both of which were statistically significant (P<0.01).

*The Medical Aid classifies beneficiaries into two categories, type 1 and 2, on the basis of being incapable (those under 18 or 65 years of age, or disabled) or capable (type 2) of working respectively in Korea.

In comparison with previous reports on subjects with a disability the reason for poor cooperation and poor management make that the therapy chosen has often been extraction by people with disability [18]. Additionally, most previous studies have overwhelmingly agreed that the disabled have poorer oral hygiene than the non-disabled [19]. And people with intellectual disability display more extensive and severe periodontal destruction and more massive colonization with periodontal pathogens compared with age-matched healthy individual [20]. The absence of oral health management continues among people with disabilities, as seen in the increase of tooth loss after school age. As a result, more people reach edentulous status prematurely. The mentally disabled had 3.09 times and the multiply disabled 4.37 times higher odds of edentulous status than did the non-disabled (Table 4) in our research. Additionally, other studies [21, 22] have also reported that adults with disabilities had more missing teeth and fewer filled teeth than the general population, but the same mean dental caries levels and high plaque levels. These results indicate people with disabilities do not receive dental treatment at the proper time. Thus, they have no choice but to extract their untreated teeth, which makes for worse oral health and eventually may lead to the deterioration of their systemic health. In this light, we need to provide policies and treatment for decreasing the number of missing teeth, such as maintenance management for people with a disability or an additional insurance system because people with disabilities develop worse oral health at a younger age than do non-disabled people. For improving dental accessibility, mobile care units are needed, as well as an administrative system such as the expansion of the existing workforce of a mobile dental care team.

**Table 4:** Logistic regression analysis to assess connection between edentulous patients and disability.

	Edentulous status		OR (95% CI)
Independent variable	Dentulous	Edentulous	
Type of disability			
Non-disabled	2,926 (99.0)	29 (1.0)	
Physically disabled	179 (96.8)	6 (3.2)	1.47 (0.44–4.93)
Mentally disabled	664 (96.1)	27 (3.9)	3.09 (1.07–8.97)
Multiply disabled	103 (94.5)	6 (5.5)	4.37 (1.16–16.37)
Gender			
Male	2,725 (97.7)	63 (2.3)	
Female	1,147 (99.6)	5 (0.4)	0.44 (0.17–1.17)
Education			
≤Elementary school	1,199 (96.1)	49 (3.9)	
Middle school	522 (98.9)	6 (1.1)	0.47 (0.19–1.14)
High school	1,778 (99.4)	10 (0.6)	0.52 (0.25–1.08)
≥College	373 (99.2)	3 (0.8)	0.58 (0.17–1.98)
Health security system*			
National health insurance	3,228 (99.0)	31 (1.0)	
Medical aid type 1	556 (93.8)	37 (6.2)	1.71 (0.59–4.97)
Medical aid type 2	88 (100.0)	0 (0.0)	0.00 (0.00)
Region			
Urban	3,027 (98.2)	57 (1.8)	
Suburban	845 (98.7)	11 (1.3)	1.29 (0.63–2.63)

Additional independent variables included in this model were age (7 categories) and income (5 categories), both of which were statistically significant (P<0.01).

*The Medical Aid classifies beneficiaries into two categories, type 1 and 2, on the basis of being incapable (those under 18 or 65 years of age, or disabled) or capable (type 2) of working respectively in Korea.

There is relatively strong evidence for an inverse relationship between socioeconomic status (SES) and the prevalence of dental caries worldwide. For example, socioeconomic status and selected behavioral determinants as risk factors for dental caries have been reported [23].Low-income whites residing in disadvantaged neighborhoods had 1.8-fold (95% confidence interval = 1.2, 2.7) higher odds of having severe periodontitis than did high-income people living in advantaged areas [24]. Many indicators of SES have been evaluated for their relationship between function and disability, but other cohorts and studies could not be excluded from the selection bias for control group selection [25]. Unhealthy and sick persons may participate less than healthy individuals, as illustrated by those receiving disability benefits. However, this selection was independent of gender, education, and region, leaving the associations between disability benefits and these sociodemographic variables unbiased [26]. Individuals with disabilities are regarded as a highly vulnerable population group, particularly as far as oral health is concern[27]. Also,the disabled have a different socioeconomic status to the non-disabled. These differences make it harder to select controls to evaluate the effect of disability on health outcomes, including oral health status [28]. In original cohort data, there are differences of socioeconomic status between the disabled and the non-disabled (Table 1). Especially, Each group have different distribution on income, education level, and type of health security system. Therefore, we used propensity score matching method of health outcome comparison data for selecting the correct controls for analyzing the net effect, which will help avoid a lack or an excess of policies in medical and dental studies [29].

The evidence of inequalities was a greater likelihood of major illness, of developing health problems at an earlier age than the rest of the population, and of dying earlier. There were many reasons for these findings; for example, poverty is linked to poorer general and inequalities in oral health, limited access to primary health care centers, and a lack of healthy cognition. In particular, it was reported that the reasons why people with disabilities did not go to the dental hospital were linked to pecuniary embarrassment (79.7%) and accessibility to the hospital (4.6%) [30].

There are various alternatives used around the world for promoting the oral health of people with a disability. In Japan, long-term preventive management for individuals with a disability has led to decrease dental caries from a younger age. Further, it is suggested that the disabled persons with severely decayed teeth in primary dentition experience more caries [31]. In addition, family dentists for persons with disabilities as part of the program for dental health services improves participants’ oral health and the quality of life of disabled persons [32]. In America, there are portable dental offices and bridge campaigns of concern, which are a dental outreach programs to help developmentally disabled individuals by bringing hygienists into schools, vocational centers, and group homes. In addition, hygienists provide preventative educational services to people with poor oral hygiene [33]. In Korea, dental treatments for people with disabilities are confined to oral health care, such as simple extractions and fillings by volunteer dentists and some of the specialized dental hospital centers.

There are also well-made regulations and policies for vulnerable social groups, especially the disabled, the poor, and the elderly, in developed countries with public welfare. Also, most of the countries implement a policy that expands public health for persons with disabilities. For strategic development, accurate data are an essential prerequisite for a problem.

Through it is hard to objectively compare in case of people with special condition like disability, our study using matched analysis on socioeconomic status between the disabled and the non-disabled. Results of our study can be used as making objective comparison to current oral health status of people with disability. We hope that this study will contribute to establishing oral health regulations that improve the health conditions of people with disabilities.

## Supporting information

S1 File STROBE checklist. (DOC)

S2 File Raw data. Original survey data was included on this file and Control group data are available from the Korea National Health and Nutrition Examination Survey database (https://knhanes.cdc.go.kr/knhanes/eng/index.do) for researchers who meet the criteria for access to confidential data.
(XLSX)
